# High capacity ammonia adsorption in a robust metal–organic framework mediated by reversible host–guest interactions†

**DOI:** 10.1039/d2cc01197b

**Published:** 2022-04-13

**Authors:** Lixia Guo, Xue Han, Yujie Ma, Jiangnan Li, Wanpeng Lu, Weiyao Li, Daniel Lee, Ivan da Silva, Yongqiang Cheng, Svemir Rudić, Pascal Manuel, Mark D. Frogley, Anibal J. Ramirez-Cuesta, Martin Schröder, Sihai Yang

**Affiliations:** Department of Chemistry, University of Manchester Manchester M13 9PL UK M.Schroder@manchester.ac.uk Sihai.Yang@manchester.ac.uk; Department of Chemical Engineering and Analytical Science, University of Manchester Manchester M13 9PL UK; ISIS Facility, STFC Rutherford Appleton Laboratory, Oxfordshire OX11 0QX UK; Neutron Scattering Division, Neutron Sciences Directorate, Oak Ridge National Laboratory Oak Ridge TN 37831 USA; Diamond Light Source, Harwell Science and Innovation Campus, Oxfordshire OX11 0DE UK

## Abstract

To understand the exceptional adsorption of ammonia (NH_3_) in MFM-300(Sc) (19.5 mmol g^−1^ at 273 K and 1 bar without hysteresis), we report a systematic investigation of the mechanism of adsorption by a combination of *in situ* neutron powder diffraction, inelastic neutron scattering, synchrotron infrared microspectroscopy, and solid-state ^45^Sc NMR spectroscopy. These complementary techniques reveal the formation of reversible host–guest supramolecular interactions, which explains directly the observed excellent reversibility of this material over 90 adsorption–desorption cycles.

## Abstract 2

To understand the exceptional adsorption of NH_3_ in MFM-300(Sc), we report a full study of the mechanism of adsorption by *in situ* neutron diffraction, inelastic neutron scattering, synchrotron infrared microspectroscopy, and ^45^Sc NMR spectroscopy.

## Graphical abstract

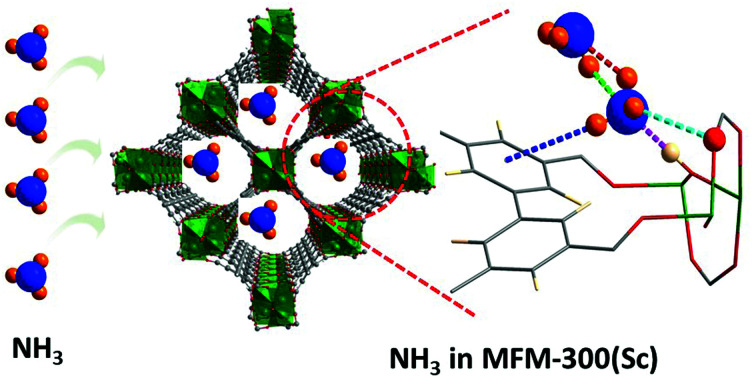

Annual global production of ammonia (NH_3_) is around 170 million tonnes reflecting its role as a major feedstock for agriculture and industry.^1^ The high hydrogen content (17.8 wt %) and hydrogen volume density (105 kg m^−3^) of NH_3_ make it a desirable carbon-free hydrogen carrier, and NH_3_ is therefore regarded as a surrogate for the H_2_ economy. However, the corrosive and toxic nature of NH_3_ makes the development of stable storage materials with high and reversible uptakes extremely challenging. Conventional sorbent materials such as zeolites,^2^ activated carbons,^3^ and organic polymers^4^ have been investigated for the storage of NH_3_, but show low and often irreversible uptakes. Metal–organic framework (MOF) materials have been postulated as promising candidates for gas storage due to their high surface areas and versatile pore structures.^5^ As opposed to conventional adsorbents, the affinities of MOF materials to a target gas can be tailored by grafting the pore interior with functional groups to anchor the gas through coordination, hydrogen bonding, electrostatic interactions, acid–base interactions or π···π stacking.^5–7^ A large number of MOFs with functional groups (*e.g.* –COOH,^8^ –OH^9^) and open metal sites^10^ have been reported to impart enhanced affinity to gas molecules. Several state-of-the-art MOFs, such as MOF-177,^11^ M_2_Cl_2_BBTA [BBTA = 1*H*,5*H*-benzo(1,2-d:4,5-*d*′)bistriazole; M = Co, Mn],^12^ M_2_Cl_2_(BTDD) {BTDD = bis(1*H*-1,2,3-triazolo[4,5-b],[4′,5′-*i*])dibenzo[1,4]dioxin; M = Mn, Co, Ni and Cu}^13^ as well as MFM-300(M) (M = Al, Fe, V, Cr, In),^6,14^ have been investigated for NH_3_ adsorption. However, due to the reactive and corrosive nature of NH_3_, many MOF systems showed structural degradation and/or significant loss of uptake after consecutive cycles owing to irreversible host–guest binding. So far, only a very limited number of MOFs exhibit reversible NH_3_ sorption over multiple cycles.^6,8,13–16^ Unravelling the molecular details of host–guest interactions is of critical importance if new efficient NH_3_ storage systems are to be developed. This is however highly challenging, not least because hydrogen atoms are invisible in X-ray diffraction experiments and NH_3_ molecules can act as a rapid rotor even in solid state.

The mechanism of adsorption of NH_3_ in MFM-300(Sc) was examined systematically using gas isotherms, breakthrough experiments, *in situ* solid-state nuclear magnetic resonance (ssNMR) spectroscopy, synchrotron infrared microspectroscopy, neutron powder diffraction (NPD) and inelastic neutron scattering (INS) techniques, coupled with DFT modelling. Distinct new insights have been gained into the mechanism of adsorption compared with a recent report based on theoretical and infrared spectroscopic studies of this system.^17^ Importantly, we found the exceptional NH_3_ uptake (19.5 mmol g^−1^ at 273 K and 1 bar) by MFM-300(Sc) was mediated by reversible host–guest and guest–guest hydrogen bond interactions. The moderate strength of the host–guest interaction in MFM-300(Sc) leads to excellent adsorption reversibility and stability with full retention of the capacity over 90 cycles.

MFM-300(Sc) shows a three-dimensional framework containing [ScO_4_(OH)_2_] octahedra which are connected *via* the *cis*-μ_2_-OH groups into infinite chains, and further coordinated by the BPTC^4−^ ligand (H_4_BPTC = biphenyl-3,3,5,5-tetracarboxylic acid) (Fig. S1, ESI†).^18^ Desolvated MFM-300(Sc) shows a Brunauer–Emmett–Teller (BET) surface area of 1390 m^2^ g^−1^ and a pore volume of 0.48 cm^3^ g^−1^ (Fig. S2, ESI†). MFM-300(Sc) exhibits high thermal stability up to 500 °C under N_2_ (Fig. S3, ESI†) and high chemical stability in aqueous solutions of pH of 7–12 as well as in various organic solvents (Fig. S4, ESI†).

Adsorption isotherms of NH_3_ for MFM-300(Sc) were measured at 273–313 K, where an exceptional uptake of 19.5 mmol g^−1^ was recorded at 273 K and 1.0 bar (Fig. 1a), reducing to 13.5 mmol g^−1^ at 298 K. MFM-300(Sc) shows the highest NH_3_ uptake among the MFM-300(M) family^6,14^ primarily due to its large pore size and pore volume allowing the accommodation of additional NH_3_ molecules in the pore. MFM-300(Sc) shows an NH_3_ uptake of 13.5 mmol g^−1^ at 298 K and 1.0 bar, comparing favourably with state-of-the-art materials (Fig. 1d and Table S6, ESI†). The uptake of NH_3_ in MFM-300(Sc) between 273 and 313 K decreases gradually with increasing temperature, consistent with an exothermic adsorption mechanism.^19^ The isosteric heat of adsorption (*Q*_st_) for NH_3_ in MFM-300(Sc) decreases from 60 to 30 kJ mol^−1^ with increasing loading of NH_3_ from 1 to 10 mmol g^−1^ (Fig. S7, ESI†), confirming the presence of moderate adsorbate–adsorbent binding interaction. The repeated isotherm of NH_3_ at 273 K using regenerated MFM-300(Sc) shows no loss in capacity with full retention of its porosity (Fig. S6, ESI†). 90 consecutive cycles of adsorption–desorption were conducted at 298 K and confirmed excellent reversibility and stability of adsorption (Fig. 1b), with retention of the crystal structure of MFM-300(Sc) as confirmed by powder X-ray diffraction (PXRD) (Fig. S5, ESI†). The ability of MFM-300(Sc) to capture NH_3_ at low concentrations (1000 ppm) was confirmed by dynamic breakthrough experiments at 298 K with a dynamic uptake of 1.65 mmol g^−1^, consistent with that measured by isotherms (1.74 mmol g^−1^ at 10 mbar, equivalent to 1000 ppm; Fig. 1c). With an exceptional adsorption capacity and excellent regenerability, MFM-300(Sc) represents a promising candidate for applications in NH_3_ storage and transport.

**Fig. 1:**
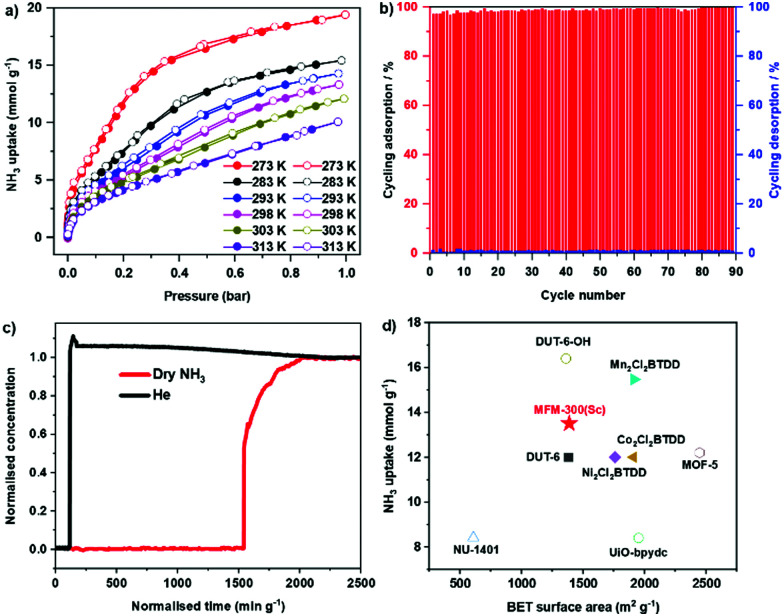
(a) Adsorption isotherms for NH_3_ in MFM-300(Sc) at 273–313 K (adsorption: solid symbols; desorption: open symbols). (b) 90 cycles of adsorption–desorption of NH_3_ in MFM-300(Sc) under pressure-swing conditions. (c) Breakthrough curve for NH_3_ (1000 ppm diluted in He) through a fixed-bed packed with MFM-300(Sc) at 298 K and 1.0 bar. (d) Comparison of NH_3_ uptake at 1 bar and 298 K for selected materials plotted against their surface areas (solid symbols: reversible sorption; hollow symbols: irreversible sorption; full details are given in the supplementary information).

*In situ* NPD data of MFM-300(Sc) as a function of ND_3_ loading were collected and Rietveld refinements revealed the preferential binding sites for ND_3_ (Fig. 2). Interestingly, the NH_3_-induced rearrangement of metal–ligand (Sc–O) bonds *via* insertion of NH_3_ molecules into the MOF upon ND_3_ binding as predicted by a DFT study^17^ was not observed here. For MFM-300(Sc)·(ND_3_)_1.25_, {[Sc_2_(L)(OD_0.6_H_0.4_)_2_]·(ND_2.05_H_0.95_)_1.25_}, only one binding site was found, interacting primarily with the bridging μ_2_-OH groups at the four corners of its square-shaped channel [O_bridge_–H⋯ND_3_ = 1.96(1) Å] (Fig. 2a and b). At the higher loading of MFM-300(Sc)·(ND_3_)_2.6_, {[Sc_2_(L)(OD_0.75_H_0.25_)_2_]·(ND_2.42_H_0.58_)_2.6_}, two distinct binding sites were identified (Fig. 2c and d). Site I is fully occupied by ND_3_ molecules (1 ND_3_/Sc), with hydrogen bonding between the μ_2_-OH groups and the ND_3_ molecule [O_bridge_–H⋯ND_3_ = 1.93(1) Å]. This is complemented by additional electrostatic interactions [ND_3_⋯aromatic rings = 3.13(1) Å], and hydrogen bonding [ND_3_⋯O_ligand_ = 3.24(1) Å]. Site II (0.3 ND_3_/Sc) exhibited hydrogen bonding with the ND_3_ at site I [2.30(3) Å and 2.24(2) Å], propagating along the 1D channel to form a cooperative {ND_3_}_∞_ network. Similar to other MFM-300 analogues,^6,14^ hydrogen/deuterium site exchange was also observed between the adsorbed ND_3_ molecules and the μ_2_-OH group for MFM-300(Sc).

**Fig. 2:**
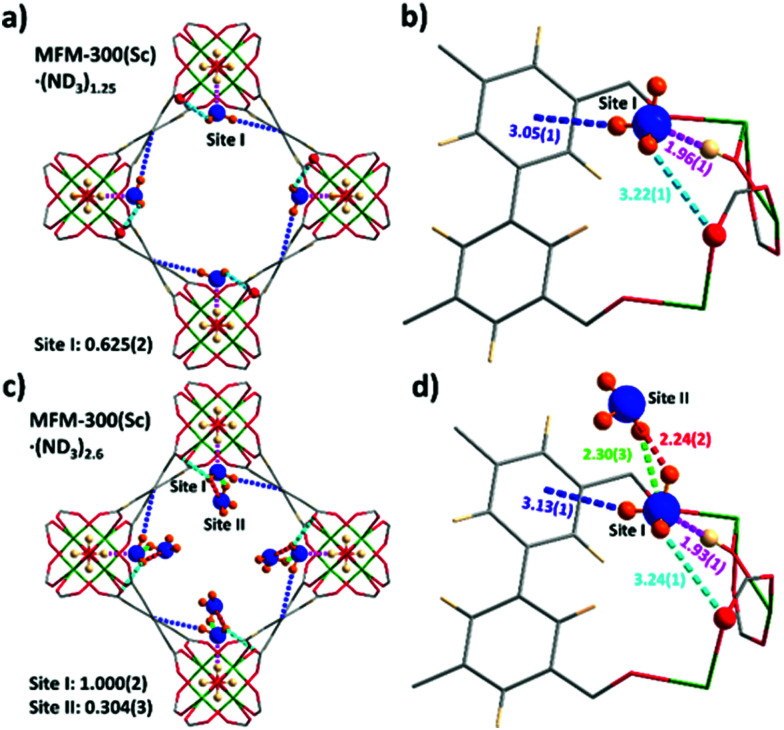
Views of binding sites for ND_3_ in MFM-300(Sc) determined by NPD at 10 K. The occupancy of each site has been converted into ND_3_/Sc for clarity. (a and c) Views along the *c*-axis showing packing of the guest molecules of ND_3_ in MFM-300(Sc)·(ND_3_)_1.25_ and MFM-300(Sc)·(ND_3_)_2.6_, respectively. (b and d) Detailed views of host–guest interactions between MFM-300(Sc) and adsorbed molecules of ND_3_.

The analysis of the NPD data were entirely consistent with information from solid-state NMR spectroscopy. Upon loading MFM-300(Sc) with NH_3_, only slight structural modifications were observed, and the crystalline nature of the framework was retained. ^45^Sc magic angle spinning (MAS) NMR spectroscopy confirmed that the geometry around the Sc(iii) centre was not notably distorted by interaction with NH_3_, with the μ_2_-OH groups (Fig. S11a, ESI†) and {^1^H-}^13^C CPMAS NMR spectra showing that the carboxyl resonance from the linker is unaffected upon NH_3_ loading (*i.e.* minimal metal site distortion). However, the resonances assigned to the aromatic carbons do shift, reflecting an interaction of the rings with the guest molecules (Fig. S11b, ESI†). The interaction of NH_3_ with the MOF was also investigated using 2D ^1^H-^45^Sc dipolar correlation (HETCOR) NMR spectroscopy (Fig. 3). The spectrum of pristine MFM-300(Sc) (Fig. 3a) shows clear cross peaks between aromatic protons (from the linker), as well as from hydroxyl protons (μ_2_-OH groups), with the Sc(iii) site, demonstrating a close proximity between these atomic environments. The corresponding spectrum of NH_3_-loaded MFM-300(Sc) is substantially different. Whilst cross peaks with aromatic protons unchanged, cross peaks with μ_2_-OH groups have moved to higher chemical shifts, indicating the presence of hydrogen-bonding, and a new weak cross peak is observed and assigned to pore-confined NH_3_ protons. *In situ* synchrotron FTIR micro-spectra were recorded at 298 K (Fig. 3e and f). The characteristic O–H stretching mode of the μ_2_-OH group is observed at 3678 cm^−1^, which reduces in intensity and broadens upon loading of NH_3_. The band at 3404 cm^−1^ is assigned to the N–H stretching of NH_3_, and this exhibits a red shift to 3390 cm^−1^.^6,14^ The bands at 1614 and 1440 cm^−1^, assigned to ν_as_(COO^−^) and ν_s_(COO^−^), respectively,^20^ show small red shifts upon adsorption of NH_3_ (Δ = 4 − 7 cm^−1^), consistent with interaction between NH_3_ and carboxylate groups. The bands between 3800 and 1400 cm^−1^ for the local framework remain unchanged upon re-activation, confirming the high structural stability of MFM-300(Sc). Thus, the ssNMR and FTIR studies verify that NH_3_ is hydrogen-bonded to the μ_2_-OH groups *via* the lone pair of electrons on nitrogen, fully consistent with the NPD analysis.

**Fig. 3:**
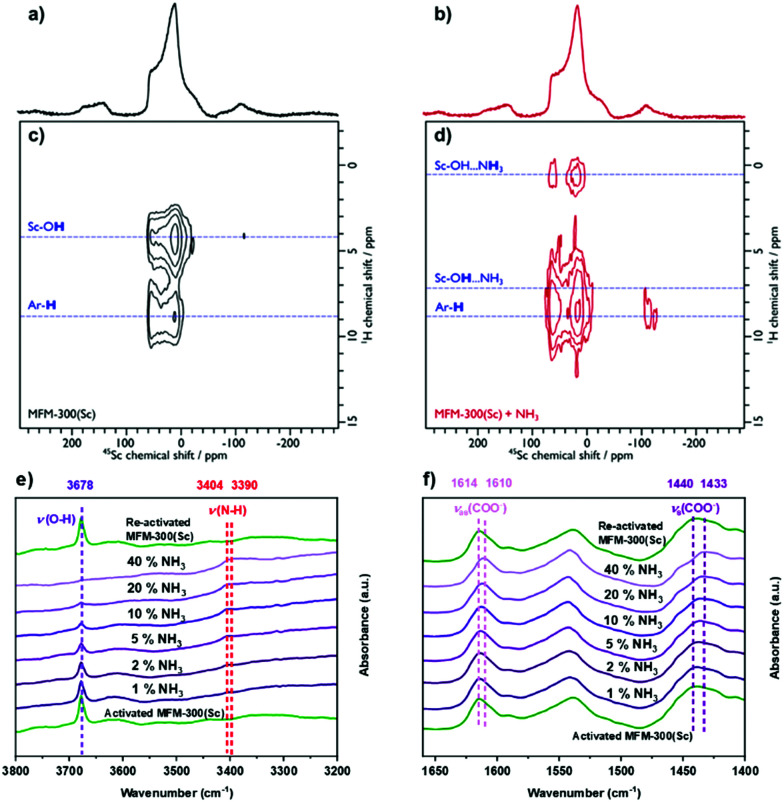
^1^H–^45^Sc Heteronuclear dipolar correlation spectroscopy (HETCOR) MAS NMR spectra of (a, c) pristine and (b, d) NH_3_-loaded MFM-300(Sc), with corresponding ^45^Sc MAS NMR spectra (top). The spectra were recorded at 9.4 T using a MAS frequency of 12 kHz. The dashed blue lines highlight correlations between the Sc(iii) site and various proton environments. *In situ* FTIR spectra of MFM-300(Sc) as a function of NH_3_ loading (diluted in dry N_2_) and re-activated under a flow of dry N_2_ at 100 mL min^−1^ at 298 K for 2 h: (e) 3800–3200 cm^−1^, (f) 1650–1400 cm^−1^.

INS spectra of bare and NH_3_-loaded MFM-300(Sc) were also collected (Fig. S12, ESI†) and simulated using DFT calculations based upon the structural models derived from NPD analyses (Fig. S13, ESI†). The difference spectra (Fig. 4a), which were obtained by subtracting the spectrum of the bare MOF from that of the NH_3_-loaded MOF, show the vibrational features of both the adsorbed NH_3_ molecules and the changes for the MOF host. The peaks in the low energy region (Fig. 4b) are primarily due to the vibrational modes of adsorbed NH_3_ molecules, with a small contribution due to changes in the lattice modes of the framework. The agreement between experimental and simulated spectra in terms of the overall profile allows unambiguous assignment of all major peaks. Specifically, the bands between 45 and 116 cm^−1^ are assigned to the translational motion of the NH_3_, which includes the vibration of NH_3_ molecules perpendicular to and along the molecular *C*_3_ axis and the hybrid of these modes. Peaks at 132 and 172 cm^−1^ are due to rotational motion of the NH_3_ around its *C*_3_ axis. Bands between 207 and 334 cm^−1^ are assigned to the rocking modes of the NH_3_. Compared to the spectrum of NH_3_ in the solid state, where each NH_3_ forms a 3D hydrogen bonding network with 6 adjacent NH_3_ molecules, bands in all regions for the adsorbed NH_3_ shift to lower energy and exhibit more broad features, indicating more dynamic environment for the adsorbed NH_3_. The features in the higher energy region mainly reflect the modes of the framework (Fig. 4c). Features I and III are due to the broadening of the peaks at 692 and 934 cm^−1^ for bare MFM-300(Sc), and are assigned to the C–H rocking out of the C_6_ plane, in-phase and anti-phase, respectively. Feature VI at higher frequency between 1090 and 1163 cm^−1^ shows reduced intensity upon adsorption of NH_3_, and is assigned to the H_ring_ rocking within the C_6_ plane. Features II and IV originate from a significant blue shift of the peak at 754 cm^−1^ in the spectrum of bare MFM-300(Sc) to 987 cm^−1^ in the spectrum of NH_3_-loaded MFM-300(Sc). This is assigned to the rocking of μ_2_-OH within the Sc–O–Sc plane. Interestingly, the features involving the motions of H_ring_ show only broadening and a decrease in intensities, while the features involving the motion of the μ_2_-OH experience changes in energy. This indicates a stronger interaction between NH_3_ and the μ_2_-OH than with H_ring_ centres. Feature V in the difference spectrum is due to the umbrella motion of adsorbed NH_3_. Thus, the combined INS and DFT study has visualised directly the host–guest binding dynamics, consistent with the reversible and high adsorption of NH_3_ in MFM-300(Sc).

**Fig. 4:**
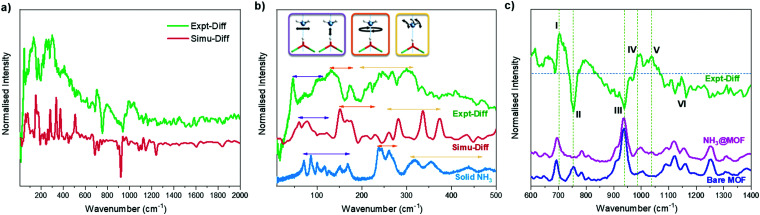
Experimental and simulated INS difference spectra of the adsorbed NH_3_ within MFM-300(Sc), denoted as Expt-Diff and Simu-Diff, respectively. (b) Comparison of the INS spectra of adsorbed NH_3_ with solid NH_3_. (c) Experimental INS spectra of bare MFM-300(Sc), NH_3_-loaded MFM-300(Sc) and the difference spectrum at the higher energy region.

In summary, MFM-300(Sc) comprised of metal-oxide chains with bridging –OH groups shows exceptional adsorption capacity (19.5 mmol g^−1^ at 273 K and 1 bar) and regenerability for NH_3_. *In situ* NPD analysis, ^45^Sc ssNMR spectroscopy, synchrotron FTIR and INS/DFT studies have unambiguously visualised the binding interactions and dynamics of NH_3_ within the pores of MFM-300(Sc). This in-depth understanding of the structure–function relationship with these host-guest systems will enable the rational design of potential materials with desired properties.

We thank EPSRC (grant EP/I011870), The Royal Society, and University of Manchester for funding. This project has received funding from the European Research Council (ERC) under the European Union's Horizon 2020 research and innovation programme (grant agreement No 742401, NANOCHEM). We are grateful to STFC/ISIS facility for access to Beamlines TOSCA and WISH. We thank Diamond Light Source for access to Beamline B22. LG and YM thank the China Scholarship Council (CSC) for funding. The computing resources were made available through the VirtuES and the ICE-MAN projects, funded by Laboratory Directed Research and Development program and Compute and Data Environment for Science (CADES) at ORNL.

## Conflicts of interest

The authors declare no competing interest.

## Supplementary Material

CC-058-D2CC01197B-s001

CC-058-D2CC01197B-s002
