# Supplementary material for: High capacity ammonia adsorption in a robust metal–organic framework mediated by reversible host–guest interactions
Source: Chem Commun (Camb). 2022 Apr 13;58(38):5753–6. doi: 10.1039/d2cc01197b (PMC9089318; doi:10.1039/d2cc01197b)
Supplement: CC-058-D2CC01197B-s001 [file CC-058-D2CC01197B-s001.pdf]

Electronic Supplementary Material (ESI) for Chemical Communications.
This journal is © The Royal Society of Chemistry 2022

Electronic Supplementary Information

High capacity ammonia adsorption in a robust metal-organic framework mediated by reversible host-guest interactions

Lixia Guo[a], Xue Han[a], Yujie Ma[a], Jiangnan Li[a], Wanpeng Lu[a], Weiyao Li[a], Daniel Lee[b], Ivan da Silva[c], Yongqiang Cheng[d], Svemir Rudić[c], Pascal Manuel[c], Mark D. Frogley[e], Anibal J. Ramirez-Cuesta[d], Martin Schröder[a]* and Sihai Yang[a]*

[a]Department of Chemistry, University of Manchester, Manchester, M13 9PL (UK)

M.Schroder@manchester.ac.uk; Sihai.Yang@manchester.ac.uk

[b]Department of Chemical Engineering and Analytical Science, University of Manchester, Manchester, M13 9PL (UK)

[c]ISIS Facility, STFC Rutherford Appleton Laboratory, Oxfordshire, OX11 0QX (UK)

[d]Neutron Scattering Division, Neutron Sciences Directorate, Oak Ridge National Laboratory, Oak Ridge, TN 37831 (USA)

[e]Diamond Light Source, Harwell Science and Innovation Campus, Oxfordshire, OX11 0DE (UK)

List of Contents

1. Experimental Section

2. Structure of MFM-300(Sc)

3. Characterisation of Porosity

4. Thermogravimetric Analysis

5. Stability Tests

6. Calculation of Isosteric Heats of Adsorption

7. Neutron Powder Diffraction

8. Solid-state NMR spectroscopy

9. Inelastic Neutron Scattering

10. Author Contribution Section

11. References

1. Experimental Section

1.1 Synthesis of MFM-300(Sc)

MFM-300(Sc) was synthesised by a solvothermal method according to the literature.[1] Scandium triflate (900 mg, 1.83 mmol) and biphenyl-3,3',5,5'-tetracarboxylic acid (H_4L, 300 mg, 0.91 mmol) were mixed in dimethylformamide (DMF, 105 mL), H_2O (15 mL) and HCl (36.5%, 3 mL). The mixture was stirred until complete dissolution occurred. The solution was then placed in a pressure tube and heated in an oil bath to 80 ^{o}C for 72 h. The tube was then cooled to room temperature, and the colourless crystalline product was separated by filtration, washed with DMF three times and stored in acetone. Yield: 70% (based on ligand).

1.2 Characterisations

Powder X-ray diffraction (PXRD) patterns was performed for the as-synthesised, post-isotherm and post-cycling experiments samples of MFM-300(Sc) on a Philips X'pert X-ray diffractometer (40 kV and 30 mA) using Cu-Kα radiation (λ = 1.5406 Å). The data were collected at room temperature in a 2θ range of 5-50 with a scan speed of 4° min^{-1}. TGA was conducted on a TA Instrument Q600 under N_2 flow of 50 mL min^{-1}. 10 mg sample was added into an alumina pan and heated from room temperature with a ramp rate of 5 °C min^{-1} up to 800 °C.

BET surface areas were obtained from N_2 isotherms recorded on a 3-flex instrument at 77 K. The pre-dried acetone-exchanged materials (100-150 mg) were loaded into a sample cell and subjected to a dynamic vacuum (1×10^{-7} mbar) at 443 K for 10 h. Measurements of static adsorption isotherms (0-1.0 bar) for NH_3 were undertaken on an IGA gravimetric sorption analyser (Hiden Isochema, Warrington, UK). NH_3 gravimetric sorption isotherms were recorded at 273, 283, 293, 298, 303, and 313 K under ultra-high vacuum produced by a turbo pumping system with the temperature controlled using a programmed water bath and furnace bath. Research-grade NH_3 was purchased from BOC and used as received. In a typical gas adsorption experiment, 40 mg of acetone-exchanged MFM-300(Sc) was loaded into the IGA system and outgassed dynamic vacuum (1×10^{-8} mbar) at 453 K for 12 h. For the cycling experiments, the pressure of NH_3 was increased from vacuum (1×10^{-8} mbar) to 200 mbar and the uptake recorded. The pressure was then reduced to regenerate the sample without heating. This cycling process was repeated 90 times.

Breakthrough experiments were conducted on a Hiden Isochema IGA-003 with ABR attachments and a Hiden Analytical mass spectrometer using a fixed-bed tube packed with 410 mg of MFM-300(Sc). The sample was activated by heating under a flow of He at 423 K for 12 h. The fixed-bed was then cooled to 298 K. A breakthrough curve was collected with a flow of 1000 ppm NH_3 diluted in He. The flow rate of the entering gas was maintained at 25 mL min^{-1}, and the concentration of NH_3 in the exhaust gas was determined by mass spectrometry and compared with the inlet concentration C_0, where C/C_0 = 1 indicates complete breakthrough.

For tests of chemical stability, 20 mg of MFM-300(Sc) was placed in a small vial and immersed under solutions of various pH values (pH = 7-12) and different organic solvents. The vial was sealed and retained at room temperature for 12 h.

The structural determination of the binding positions of ND_3 in MFM-300(Sc) was conducted using WISH, a long wavelength powder and single crystal neutron diffractometer at the ISIS neutron and muon facility at Rutherford Appleton Laboratory (UK). The instrument has a solid methane moderator providing a high flux of cold neutrons with a large bandwidth, transported to the sample via an elliptical guide. The WISH system of divergence jaws allows tuning of the resolution according to the need of the experiment; in this case, it was setup in high resolution mode. The WISH detectors are 1m long, 8mm diameter pixelated 3He tubes positioned at 2.2m from the sample and arranged on a cylindrical locus covering a 2θ scattering angle of 10-170°. To reduce the background from the sample environment, WISH was equipped with an oscillating radial collimator that defines a cylinder of radius of approximately 22 mm diameter at 90° scattering. The sample of desolvated MFM-300(Sc) was loaded into a cylindrical vanadium sample container with an indium vacuum seal connected to a gas handling system. The sample was degassed at $1x10^{-7}$ mbar and at 100 °C for 4 days with He flushing to remove any remaining trace of guest water. The sample was dosed with ND_3 using the volumetric method after being warned to room temperature to ensure that the gas is well dispersed throughout the crystalline structure of MFM-300(Sc). Data collection for desolvated MFM-300(Sc) and two subsequent loadings of ND_3 (0.625 and 1.3 ND_3 molecules per OH functionality) were performed controlled using a He cryostat (10 ± 0.2 K).

In situ synchrotron infrared micro-spectroscopy experiments were carried out at multimode infrared imaging and micro spectroscopy (MIRIAM) beamline at the Diamond Light Source, UK. The instrument is comprised of a Bruker Hyperion 3000 microscope in transmission mode, with 15x objective and condenser lenses and a small element (50 μm) liquid N_2 cooled MCT detector, coupled to a Bruker Vertex 80 V Fourier Transform IR interferometer using radiation generated from a bending magnet source. Spectra were collected (512 scans) in the range 400-4000 cm^{-1} at 4 cm^{-1} resolution and an infrared spot size at the sample of approximately 15 × 15 μm. A microcrystalline powder of MFM-300(Sc) was scattered onto a 0.5 mm thick ZnSe infrared window and placed within a Linkam FTIR 600 gas-tight sample cell equipped with 0.5 mm thick ZnSe windows, a heating stage and gas inlet and outlet. Ultrapure N_2 and anhydrous NH_3 gases were used as supplied from the cylinder. The gases were flowed through the gas delivery system prior to connection to the Linkam cell to remove air and moisture. The gases were dosed volumetrically to the sample cell using mass flow controllers, and the total flow rate being maintained at 100 mL min^{-1} for all experiments. The exhaust from the cell was directly vented to an extraction system and the total pressure in the cell was therefore 1 bar for all experiments. The sample was desolvated under a flow of dry N_2 at 100 mL min^{-1} and 443 K for 5 h, and was then cooled to 298 K under a continuous flow of N_2. Dry NH_3 was then dosed as a function of partial pressure, maintaining a total flow of 100 mL min^{-1}. The MOF sample was regenerated with a flow of dry N_2.

Magic angle spinning (MAS) NMR spectra were recorded using a Bruker 9.4 T (400 MHz 1H Larmor frequency) AVANCE III spectrometer equipped with a 4 mm HFX MAS probe. Samples were treated and packed into 4 mm o.d. zirconia rotors under inert conditions and sealed with a Kel-F rotor cap. Experiments were acquired at ambient temperature using a MAS frequency of 12 kHz. ^{1}H-pulses of 100 kHz were used for excitation and SPINAL-64[2] heteronuclear decoupling, ^{45}Sc-pulses of 0.5 μs duration (small flip angle with radio

frequency field amplitude of ~70 kHz) were employed for ^{45}Sc direct excitation (DE)MAS experiments, and ^{13}C-pulses and spin-locking at 50 kHz were used for {^{1}H-}^{13}C CPMAS experiments with corresponding ramped (70-100%) ^{1}H spin-locking at ~73 kHz (100%) for 2 ms; s Hahn-echo τ_r–π–τ_r sequence of 2 rotor periods total duration was applied to ^{13}C after CP to circumvent receiver dead-time. For the ^{1}H-^{45}Sc 2D CP (HETCOR) dipolar correlation experiments, 500 μs of CP spin-locking was applied with fixed-amplitude RF irradiation of ~28 kHz for ^{1}H and ~4 kHz for ^{45}Sc. 24 complex t_1 increments were acquired with an indirect dimension dwell time of 83.33 μs, and spectral deconvolution and peak fitting were performed in the solid lineshape analysis (SOLA) module v2.2.4 in Bruker TopSpin v4.0.9. ^{1}H and ^{13}C chemical shifts are given with respect to TMS (0 ppm) and ^{45}Sc chemical shifts are referenced to a 0.06 M $Sc(NO_3)_3$ in D_2O solution.

INS spectra were collected on the TOSCA beamline at ISIS Neutron and Muon Source (UK). The sample of desolvated MFM-300(Sc) was loaded into a cylindrical vanadium sample container with an indium vacuum seal and this was connected to a gas handling system. The sample was degassed at 393 K and 10^{-7} mbar for 24 h to remove any residual trace of guest water. The temperature during data collection was controlled using a closed cycle refrigerator cryostat (10 ± 0.1 K). The loading of NH_3 was performed volumetrically at room temperature, and subsequently the temperature was reduced to 10 K in order to minimize achievable thermal motion of the framework and adsorbed NH_3 molecules in the scattering measurements. Background spectra of MFM-300(Sc) were subtracted to obtain the difference spectra.

Modelling by Density Functional Theory (DFT) of the bare and NH_3-loaded MFM-300(Sc) was performed using the Vienna Ab initio Simulation Package (VASP).[3] The calculation used Projector Augmented Wave (PAW) method[4,5] to describe the effects of core electrons, and Perdew-Burke-Ernzerhof (PBE)[6] implementation of the Generalized Gradient Approximation (GGA) for the exchange-correlation functional. Energy cutoff was 800 eV for the plane-wave basis of the valence electrons. The lattice parameters and atomic coordinates determined by neutron powder diffraction in this work were used as the initial structure. The electronic structure was calculated on the Γ-point for the unit cell (144 atoms for the blank MOF). The total energy tolerance for electronic energy minimization was 10^{-8} eV, and for structure optimization it was 10^{-7} eV. The maximum interatomic force after relaxation was below 0.001 eV/Å, and the optB86b-vdW functional for dispersion corrections was applied.[7] The vibrational eigen-frequencies and modes were then calculated by solving the force constants and dynamical matrix using Phonopy.[8] The OClimax software was used to convert the DFT-calculated phonon results to the simulated INS spectra.[9]

2. Structure of MFM-300(Sc)

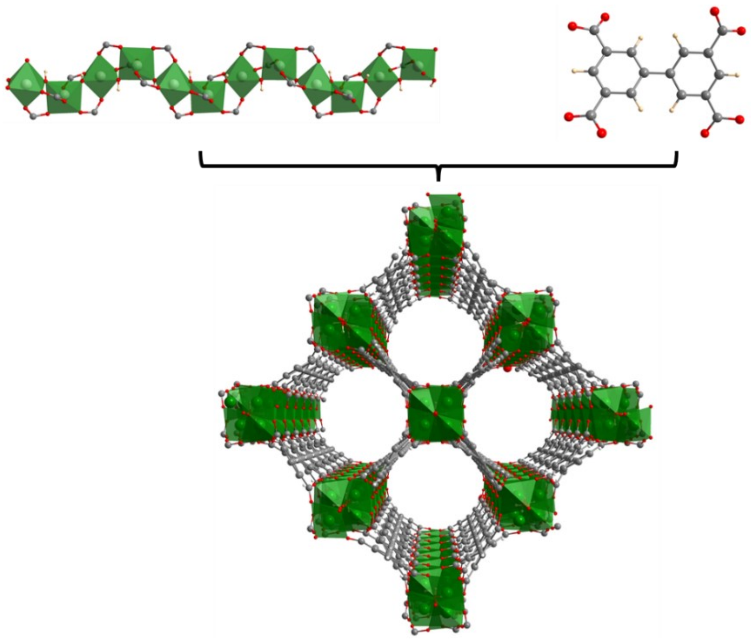

Fig. S1. View of the three-dimensional framework structure of MFM-300(Sc). Colour code for atoms: Sc, green; O, red; C, grey; H, tan; N, blue.

3. Characterisation of Porosity

Prior to the measurement, MFM-300(Sc) was activated under dynamic vacuum at 180 °C for 12 h. The adsorption-desorption isotherms for N_2 were carried out at 77 K. Fig. S2 shows a type-I profile with a surface area of 1390 $m^2\ g^{-1}$.

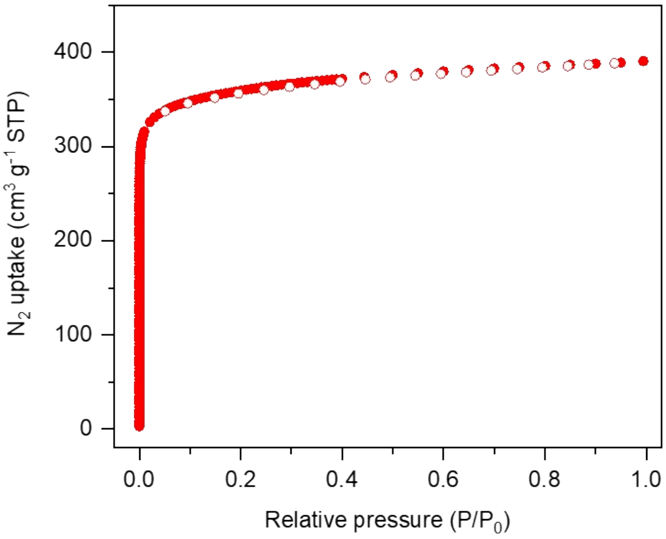

Fig. S2. Adsorption isotherm for N_2 in MFM-300(Sc) at 77 K.

4. Thermogravimetric Analysis

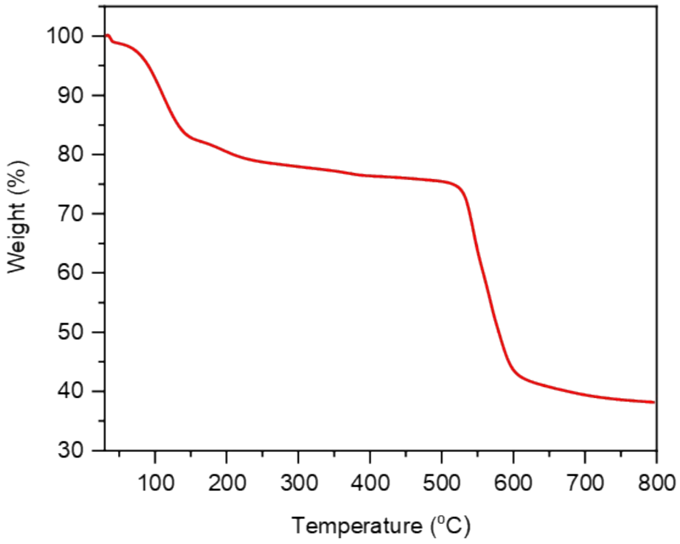

Fig. S3. Thermogravimetric analysis of acetone-exchanged MFM-300(Sc) under a flow of N_2 at a heating rate of 5 °C min^{-1}.

5. Stability Tests

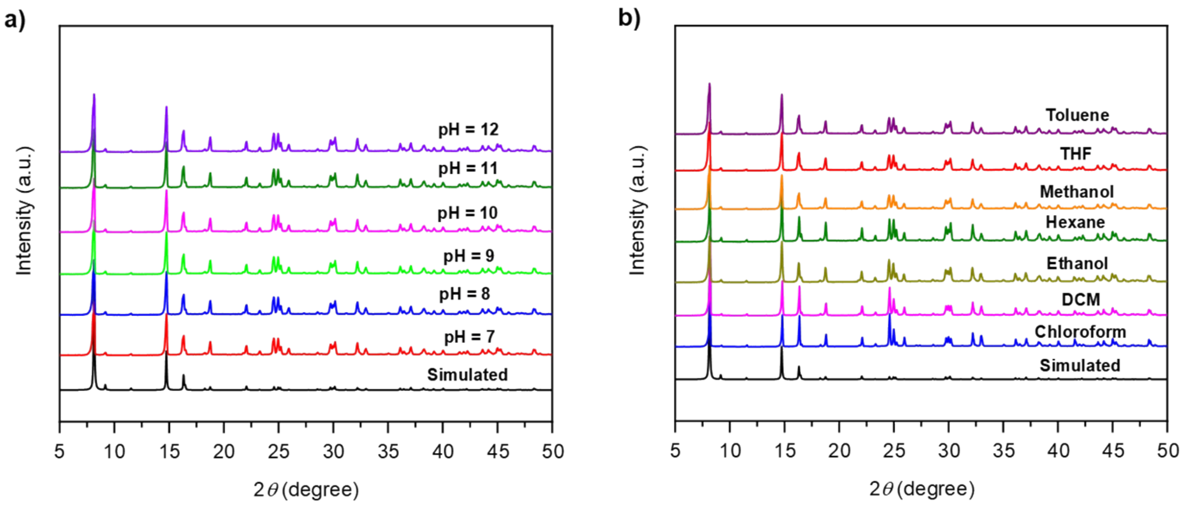

Fig. S4. PXRD patterns of MFM-300(Sc) after being immersed in (a) solutions at pH 7 to pH12, and (b) different organic solvents.

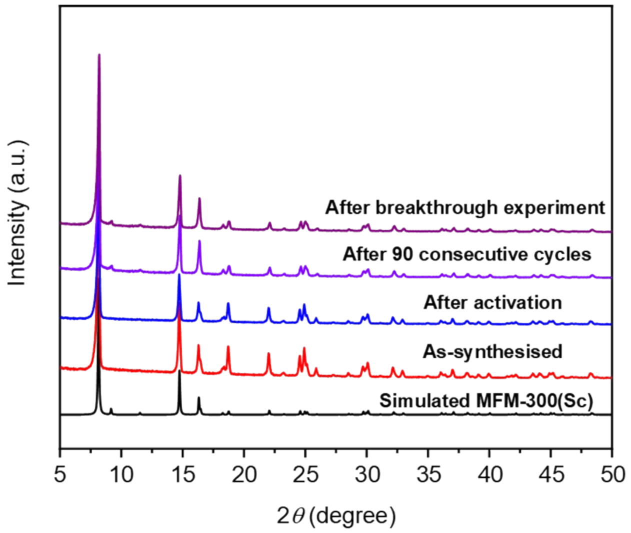

Fig. S5. PXRD patterns of as-synthesised MFM-300(Sc), activated sample, and samples after 90 consecutive cycles of adsorption of NH_3 and after NH_3 breakthrough experiment.

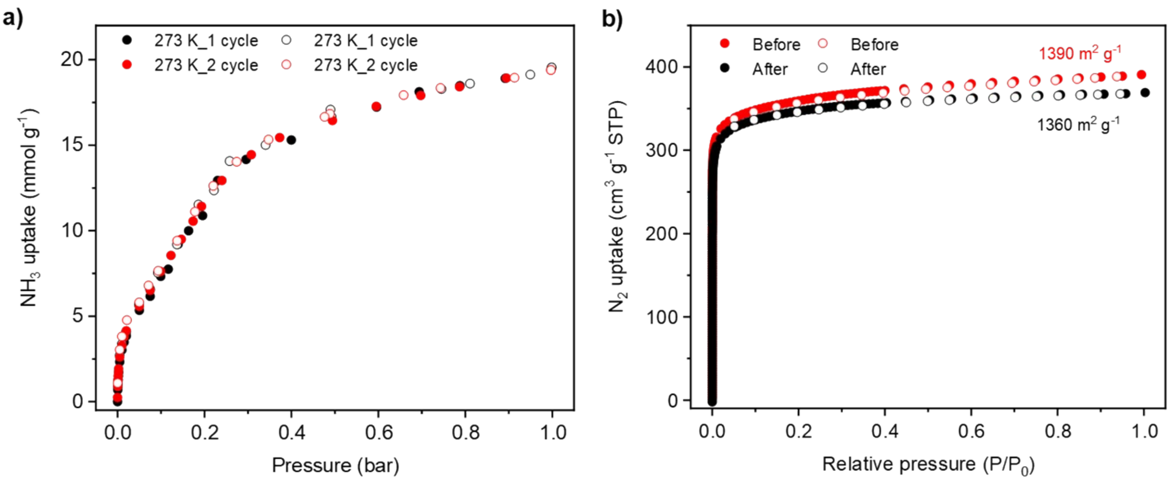

Fig. S6. (a) Adsorption isotherms for NH_3 in MFM-300(Sc) at 273 K, first cycle (black) and second cycle (red). (b) Adsorption isotherms for N_2 at 77 K in MFM-300(Sc) before and after two cycles NH_3 adsorption.

6. Calculation of Isosteric Heats of Adsorption

To calculate the differential enthalpies (ΔH_n) and entropies of adsorption (ΔS_n) for NH_3 uptake as a function of gas adsorption (n), isotherms were measured over a range of temperatures and fitted to the van't Hoff isochore (Equation 1).

$$\ln (p)_n = \frac{\Delta H_n}{RT} - \frac{\Delta S_n}{R} \quad (1)$$

Liner fitting of the plot of ln(p) versus 1/T at constant gas loading allows the isosteric heats of adsorption (Q_{st}) and entropies of adsorption (ΔS_n) to be determined from the slope and the intercept of the line, respectively.

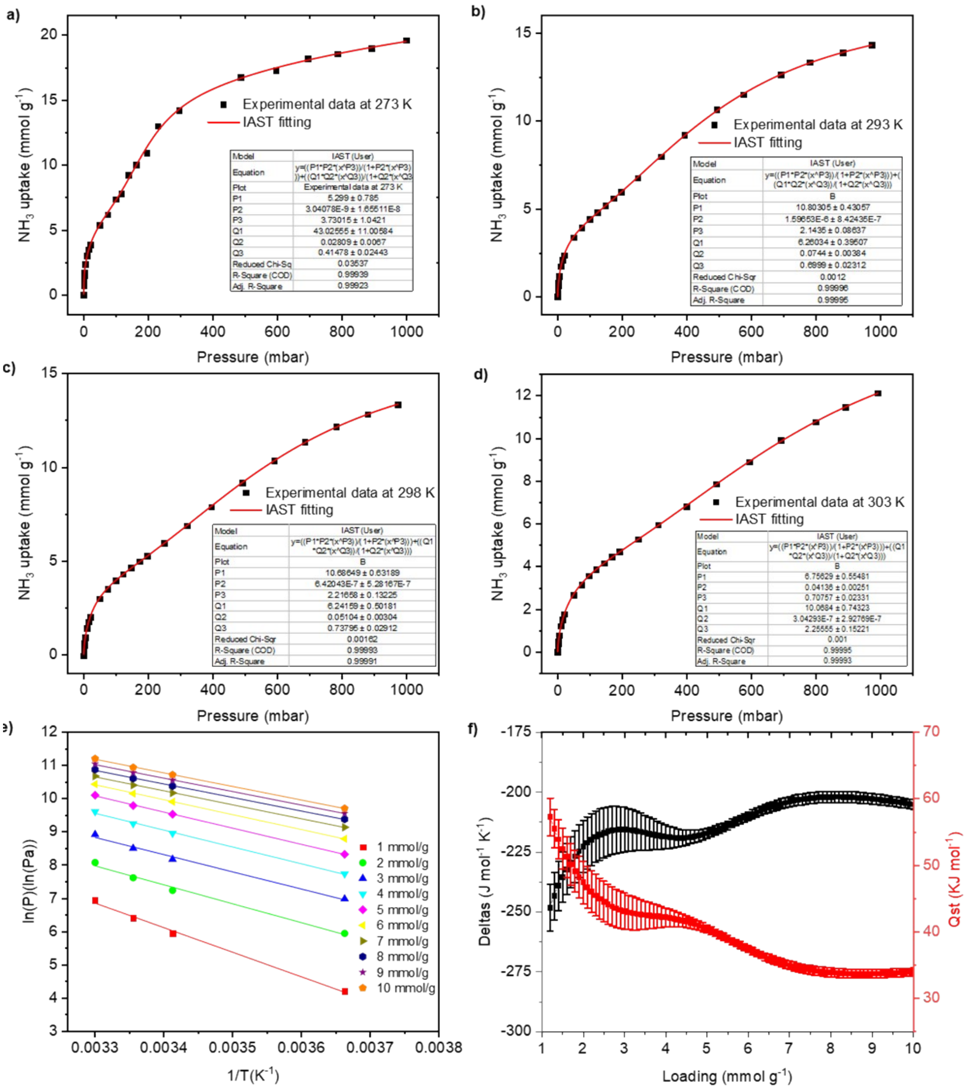

Fig. S7. (a-d) IAST fitting of isotherms for NH_3-loaded MFM-300(Sc) at different temperatures and up to 1 bar. (e) van't Hoff linear fittings and (f) isosteric enthalpy and entropy of adsorption for NH_3 in MFM-300(Sc) at different loadings.

7. Neutron Powder Diffraction

Rietveld refinements of the NPD patterns of the bare MOF and samples at various ND_3 loadings were performed using the TOPAS software package. In this treatment the guest molecules are treated as rigid bodies; we first refined the centres of mass, orientations, and occupancies of the adsorbate, followed by full profile Rietveld refinement, including the positions of metals and linkers together with their corresponding lattice parameters, resulting in satisfactory R-factors. The final refinements on all parameters including fractional coordinates, thermal parameters, occupancies for both host lattice and adsorbate molecule, and background/profile coefficients yielded very good agreement factors.

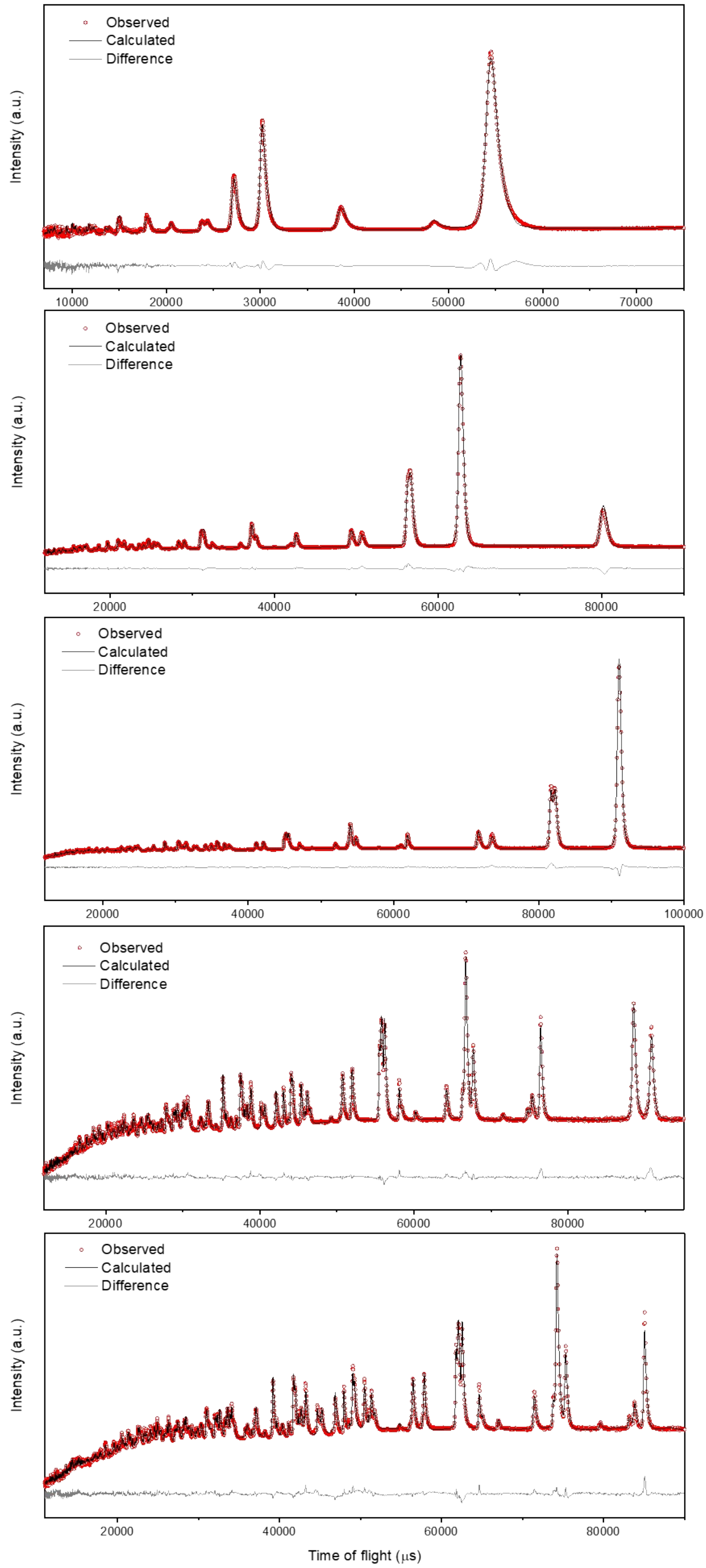

Fig. S8. Neutron diffraction patterns and Rietveld refinement of bare MFM-300(Sc) (bank 1 to 5).

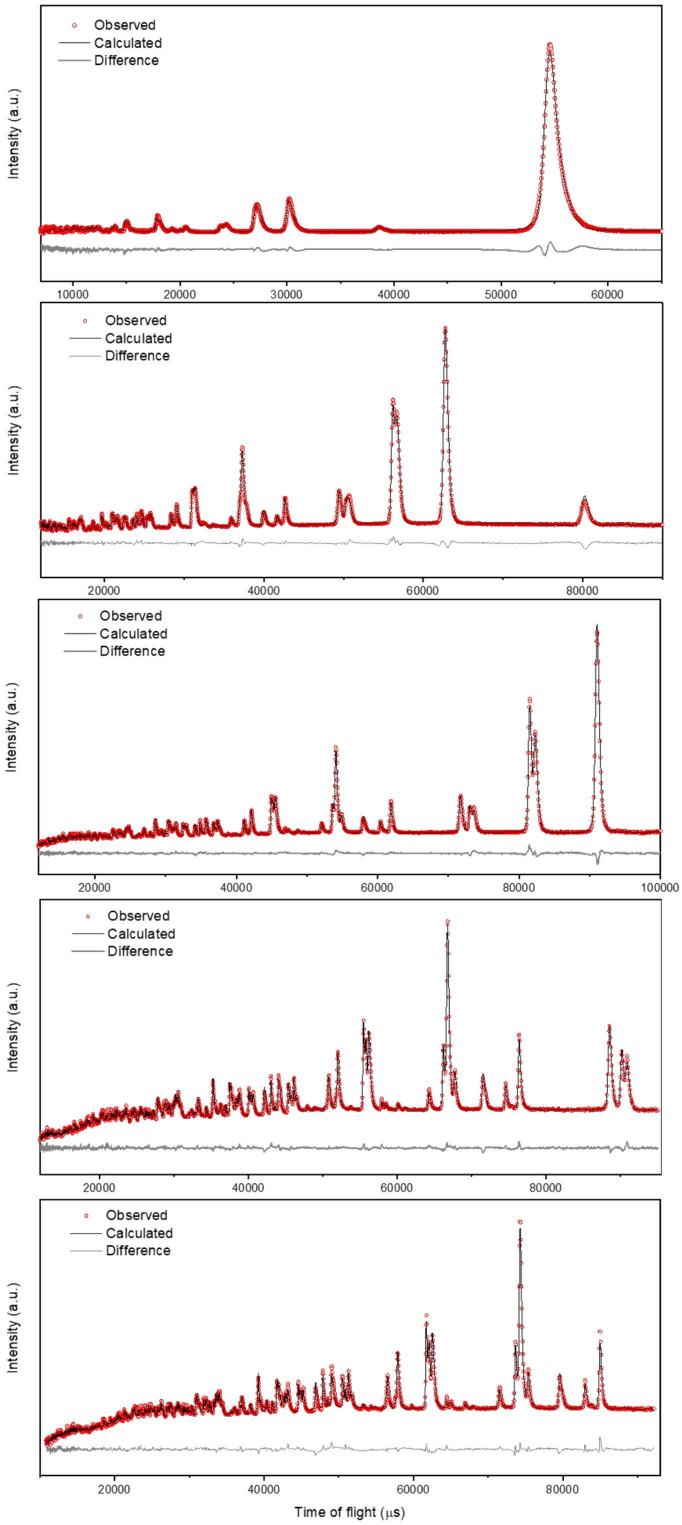

Fig. S9. Neutron diffraction patterns and Rietveld refinement of MFM-300(Sc)·$(ND_3)_{1.25}$. (bank 1 to 5).

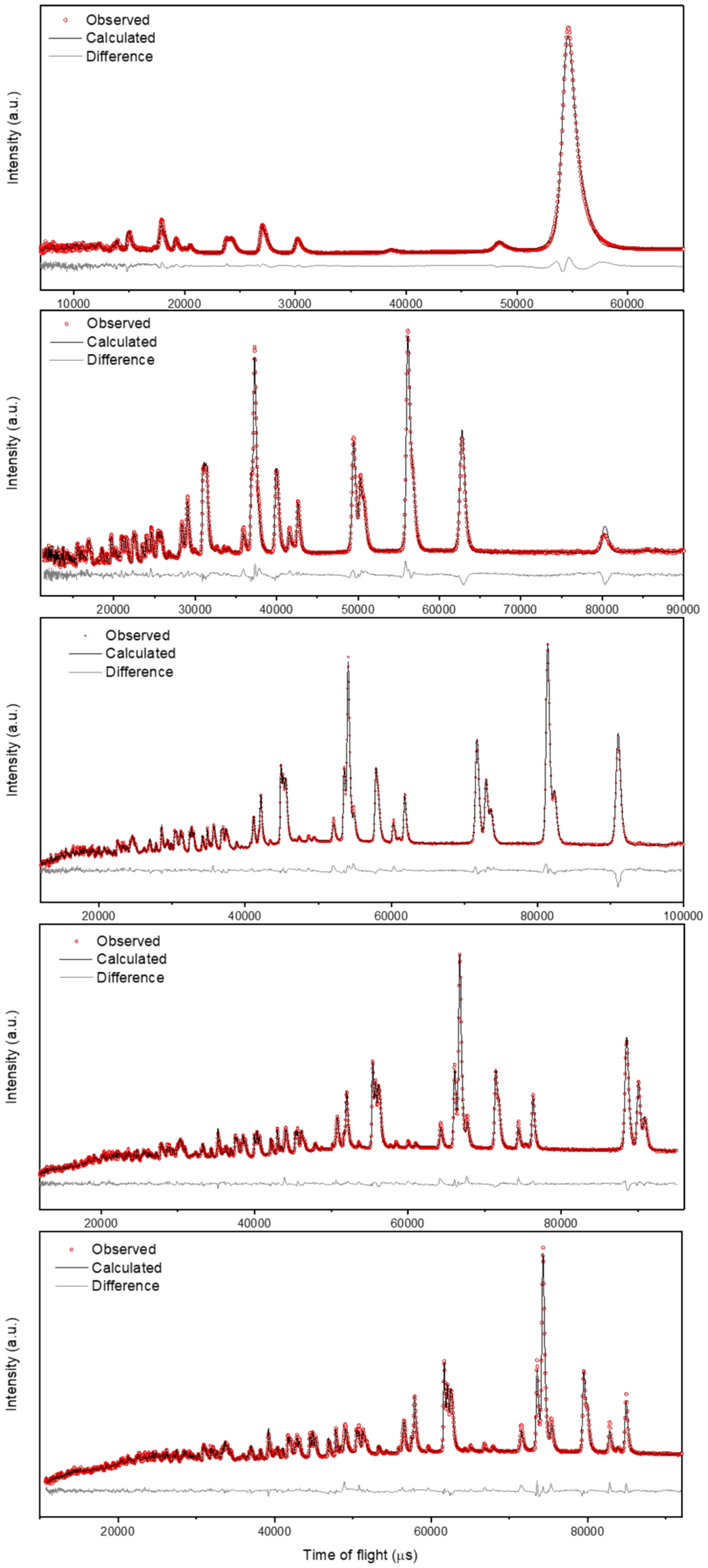

Fig. S10. Neutron diffraction patterns and Rietveld refinement of MFM-300(Sc)·$(ND_3)_{2.6}$. (bank 1 to 5).

Table S1. Crystal Data and Details of the Structure Determination for ND_3 loaded MFM-300(Sc).

	MFM-300(Sc)	MFM-300(Sc)·$(ND_3)_{1.25}$	MFM-300(Sc)·$(ND_3)_{2.6}$
Formula	$C_8H_4ScO_5$	$C_8H_4D_{1.9}ScO_5N_{0.6}$	$C_8H_4D_{3.9}ScO_5N_{1.3}$
Formula weight	225.07	237.5	251.21
Crystal system	Tetragonal	Tetragonal	Tetragonal
Space Group	$I4_122$	$I4_122$	$I4_122$
a, b (Å)	15.3819(3)	15.3970(3)	15.4020(3)
c (Å)	12.4454(2)	12.4104(2)	12.3825(3)
Volume(Å^3)	2944.6(12)	2942.1(13)	2937.4(14)
ρ (calc) g/cm^3	1.073	1.073	1.136
Radiation type	Neutron	Neutron	Neutron
Scan method	Time of flight	Time of flight	Time of flight
Temperature	10 K	10 K	10 K
R_{exp} (%)	0.41	0.37	0.36
R_{wp} (%)	1.54	1.54	1.54
R_p (%)	1.42	1.35	1.39
GoF (χ^2)	3.77	4.15	4.34
CCDC	2142629	2142631	2142630

Table S2. Host–Guest Interactions in MFM-300(Sc)·$(ND_3)_{1.25}$.

MFM-300(Sc)·$(ND_3)_{1.25}$	Interactions	Distance (Å)	Colour
Site	H (HO-Sc)···N (site I)	1.96(1)	pink
	D (site I)···Benzene	3.05(1)	blue
	D (site I)···O (ligand)	3.22(1)	turquoise

Table S3: Atomic positions for atoms in MFM-300(Sc)·$(ND_3)_{1.25}$.

	x	y	z	Occupancy	Biso / Å^2
Sc	0.6876(1)	0.3124 (1)	0.5	1	1.91(7)
O1	0.7437(3)	0.25	0.625	1	0.50(6)
O2	0.6061(4)	0.3751(4)	0.6148(3)	1	0.50(6)
O3	0.5969(4)	0.2897(2)	0.7580(3)	1	0.50(6)
C1	0.5827(3)	0.3611(2)	0.7109(2)	1	1.05(5)
C2	0.5372(1)	0.4302 (1)	0.7699(2)	1	1.05(5)
C3	0.5	0.5	0.7132(2)	1	1.05(5)
C4	0.5372(10)	0.4302(1)	0.8832(1)	1	1.05(5)
C5	0.5	0.5	0.9399(1)	1	1.05(5)
H1	0.8021(7)	0.25	0.625	0.406(3)	0.50(6)
D1	0.8021(7)	0.25	0.625	0.594(3)	0.50(6)
H3	0.5	0.5	0.6286(4)	1	1.05(5)
H4	0.5650(2)	0.3781(2)	0.9255(2)	1	1.05(5)
N1	0.75	0.0706(3)	0.375	0.625(2)	12.8(3)
D1	0.6891	0.0457(3)	0.375	0.2135(13)	12.8(3)
D2	0.7804	0.0457(3)	0.3096	0.2135(13)	12.8(3)
D3	0.7804	0.0457(3)	0.4404	0.2135(13)	12.8(3)
H1	0.6891	0.0457(3)	0.375	0.0990(5)	12.8(3)
H2	0.7804	0.0457(3)	0.3096	0.0990(5)	12.8(3)
H3	0.7804	0.0457(3)	0.4404	0.0990(5)	12.8(3)

Table S4. Host–Guest Interactions in MFM-300(Sc)·$(ND_3)_{2.6}$.

MFM-300(Sc)·$(ND_3)_{2.6}$	Interactions	Distances (Å)	Colour
Site I	H (HO-Sc)···N (site I)	1.93(1)	pink
	N (site I)···D (site II)	2.30(3)	bright green
	D (site I)···Benzene	3.13(1)	blue
	D (site I)···N (site II)	2.24(2)	red
	D (site I)···O(ligand)	3.24(1)	turquoise
Site II	N (site II)···D (site I)	2.24(2)	red
	D (site II) ···N (site I)	2.30(3)	bright green

Table S5: Atomic positions for atoms in MFM-300(Sc)·$(ND_3)_{2.6}$.

	x	y	z	Occupancy	Biso / Å^2
Sc	0.6895(1)	0.3105(2)	0.5	1	2.37(8)
O1	0.7452(4)	0.25	0.65	1	0.65(6)
O2	0.6072(4)	0.3755(4)	0.6130(3)	1	0.65(6)
O3	0.5972(4)	0.2894(3)	0.7583(4)	1	0.65(6)
C1	0.5839(3)	0.3613(2)	0.7102(2)	1	1.32(5)
C2	0.5394(2)	0.4315(1)	0.7698(2)	1	1.32(5)
C3	0.5	0.5	0.7131(3)	1	1.32(5)
C4	0.5394(2)	0.4315(1)	0.8833(2)	1	1.32(5)
C5	0.5	0.5	0.9401(2)	1	1.32(5)
H1	0.8080(6)	0.25	0.625	0.254(4)	0.65(6)
D1	0.8080(6)	0.25	0.625	0.746(4)	0.65(6)
H3	0.5	0.5	0.6283(4)	1	1.32(5)
H4	0.5688(2)	0.3803(2)	0.9257(2)	1	1.32(5)
N2	0.2636(5)	0.4537(9)	0.6766(10)	0.1522(11)	7.83(16)
D1	0.2721(15)	0.4110(16)	0.7377(16)	0.1522(11)	7.83(16)
D2	0.3085(17)	0.439(2)	0.6198(18)	0.1522(11)	7.83(16)
D3	0.280(3)	0.5130(13)	0.706(2)	0.1522(11)	7.83(16)
N1	0.75	0.0669(18)	0.375	1.000(2)	7.83(16)
D1	0.6892	0.0421(2)	0.375	0.3757(14)	7.83(16)
D2	0.7804	0.0421(2)	0.3095	0.3757(14)	7.83(16)
D3	0.7804	0.0421(2)	0.4405	0.3757(14)	7.83(16)
H1	0.6892	0.0421(2)	0.375	0.3757(14)	7.83(16)
H2	0.7804	0.0421(2)	0.3095	0.1243(7)	7.83(16)
H3	0.7804	0.0421(2)	0.4405	0.1243(7)	7.83(16)

8. Solid-state NMR spectroscopy

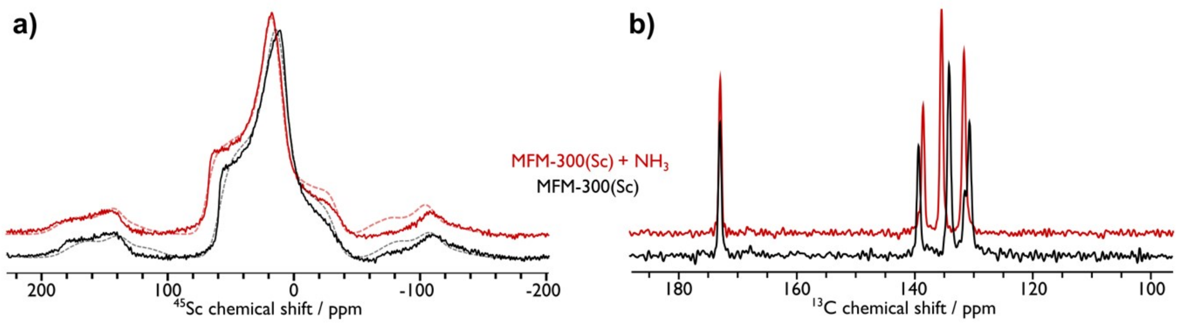

Fig. S11. (a) ^{45}Sc and (b) {^{1}H-}^{13}C CP MAS NMR spectra of pristine (black) and NH_3-loaded (red) MFM-300(Sc). The dashed lines in (a) are from simulated spectra with the following parameters: δ_{iso} = 59.6 ppm, C_Q = 10.8 MHz, η_Q = 1, Gaussian broadening = 1.41 kHz for pristine MFM-300(Sc) and δ_{iso} = 68.6 ppm, C_Q = 11.5 MHz, η_Q = 1, Gaussian broadening = 1.14 kHz for NH_3-loaded MFM-300(Sc).

9. Inelastic neutron scattering

INS was applied to study the binding interaction and structure dynamics in this case because of its several unique advantages:

(a) INS spectroscopy is ultra-sensitive to the vibrations of hydrogen atoms, which is ten times more visible than other elements due to its high neutron cross-section.

(b) The technique is not subject to any optical selection rules, neither restricted to the centre of the Brillouin zone (gamma point). All vibrations are active and, in principle, measurable.

(c) INS spectra can be readily and accurately modelled: the intensities are proportional to the concentration of elements in the sample and their cross-sections, and the measured INS intensities relate straightforwardly to the associated displacements of the scattering atom. Treatment of background correction is also relatively straightforward.

(d) Neutrons penetrate deep into materials and pass readily through the walls of metal containers making neutrons ideal to measure bulk properties of materials.

(e) INS spectrometers cover the whole range of the molecular vibrational spectrum, 0–500 meV (0-4000 cm^{-1}).

(f) Calculation of the INS spectra by DFT vibrational analysis can be readily achieved, and DFT calculations relate directly to the INS spectra, and, in the case of solid-state calculations, there are no approximations other than the use of DFT eigenvectors and eigenvalues to determine the spectral intensities. Assignments of features on the experimental difference spectra are described below.

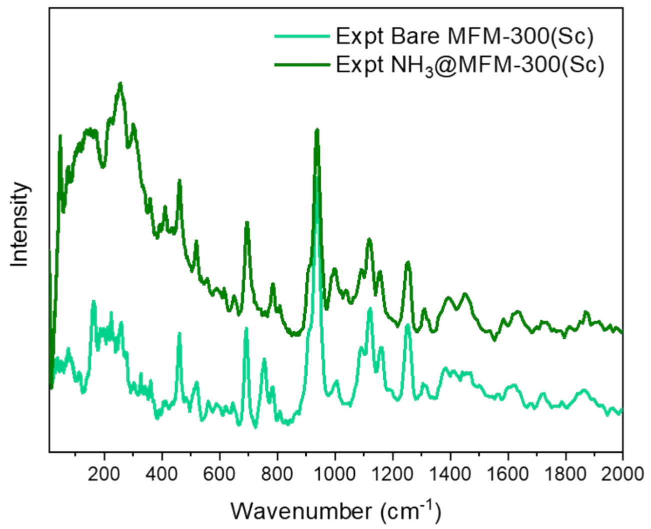

Fig. S12. Experimental INS spectra of bare MFM-300(Sc) and of NH_3-loaded MFM-300(Sc).

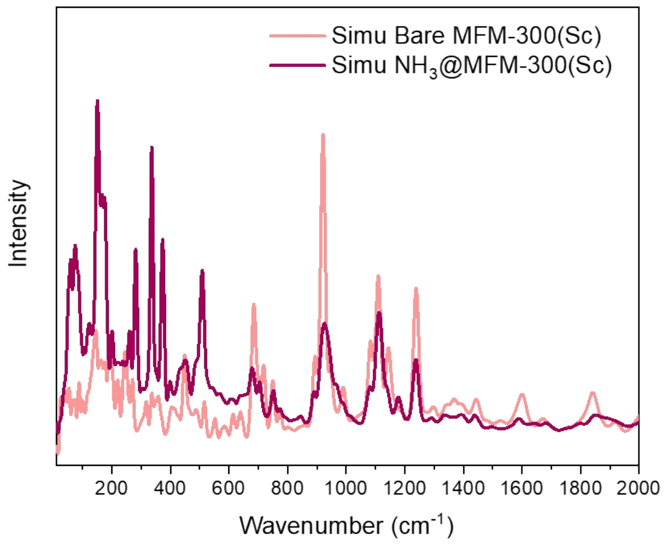

Fig. S13. Simulated INS spectra of bare MFM-300(Sc) and NH_3-loaded MFM-300(Sc).

Table S6. Comparison of the total uptake of NH_3 in selected MOF materials at 1 bar.

MOF	Capacity (mmol/g)	Structure	Regeneration condition	Stability	Reference
**MOF-5**<br>**MOF-177**	12.2 (298 K)	-	-	loss of crystallinity after adsorption	10
DUT-6<br>**OH-DUT-6**	12, 16.4 (298 K)	hydroxyl functionality	-	irreversible adsorption and structural degradation	11
**Mg-MOF-74**	16.2 (298 K)	Open metal sites	-	capacity reduced after first ads/des cycle	12
**Fe-MIL-101-SO_3H**	17.8 (298 K)	Brønsted acid	-	-	13
M_2Cl_2BTDD[a]<br>M=Mn,Co,Ni	15.5, 12.0, 12.0 (298 K)	Open metal sites	200 °C, vacuum	reversible for three ads/des cycles	14
**MIL-101**	10 (298K)	-	25 °C, 30 min, vacuum	uptake maintained for five ads/des cycles	15
M_2Cl_2BBTA[b]<br>M=Cu,Co,Ni	19.8, 18.0, 14.7 (298 K)	Open metal sites	-	Ni_2Cl_2(BBTA) retains crystallinity upon exposure	16
**NU-300**	8.28 (298 K)	Free carboxylate group	vacuum/RT	crystallinity decreases	17
**NU-1401**	8.41 (298 K)	hydroxyl groups	-	hysteresis loops between ad/desorption	18
**MFU-4**	17.7 (298 K)	hydroxyl groups	-	-	19
M_2(dobpdc)[c]<br>M=Mg,Ni,Zn, Co,Mn	23.9, 20.8, 15.2, 13.3, 13.3 (298 K)	Open metal sites	-	Mg_2(dobpdc) shows five reversible ads/des cycles under wet conditions	20
UiO-67<br>**UiO-bpydc[d]**	~8.4 (298 K)	hydroxyl groups	-	Hysteresis loop	21
MFM-300(M)<br>M=Al,Cr,V^{III},Fe, V^{IV}	15.7, 14.0, 15.6, 16.1, 17.3 (273 K)	bridging hydroxyl	-	MFM-300(Al,Cr,Fe V^{III}) shows three reversible ads/des cycles	22, 23
MFM-300(Sc)	13.1 (298 K)	Sc-N interactions		4 % of NH_3 uptake capacity reduced after 5 adsorption cycles	24
MFM-303	9.9 (273 K)	free carboxylate and hydroxyl groups	-	Keep crystalline after cycles experiment	25
UiO-67<br>Uio-67-vac<br>**UiO-67-ox-Cu**	6.1-10.5 (298 K)	accessible carboxylate group and copper sites	-	UiO-67 and UiO-67-ox-Cu missed about 28.32% and 44.6% of their surface area after five NH_3 recycles.	26

**MFM-300(Sc)**	19.5 (273 K)<br>13.5 (298 K)	bridging hydroxyl groups	-	MFM-300(Sc) shows at least two reversible ads/des cycles and keep crystalline after 90 cycles experiment	**This work**

Note: a) BTDD = bis(1*H*-1,2,3-triazolo[4,5-b],[4′,5′-*i*])dibenzo[1,4]dioxin; b) BBTA = 1*H*,5*H*-benzo(1,2-d:4,5-*d′*)bistriazole; c) dobpdc = 4,4-dioxidobiphenyl-3,3-dicarboxylate; d) bpydc = 2,2′-bipyridine-5,5′-dicarboxylate

10. Author Contribution Section

L.G. synthesised and characterised the MOF samples, measured and analysed adsorption isotherms and the breakthrough data. Y.M., X.H., Y.C., W. Li, I. S., S. R., P. M., A.R.C., collected and analysed the neutron powder diffraction and inelastic neutron scattering data. D. L. collected and analysed the ssNMR data. J.L., W. Lu and M. F. collected and analysed the IR data. M.S. and S.Y. directed and supervised the project. L.G., X.H., D.L., M.S. and S.Y prepared the manuscript.

11. References

1. X. Zhang, I. Silva, H. Godfrey, S. Callear, S. Sapchenko, Y. Cheng, I. Yrezabal, M. Frogley, G. Cinque, C. Tang, C. Giacobbe, C. Dejoie, S. Rudic, A. Cuesta, M. Denecke, S. Yang, M. Schröder, *J. Am. Chem. Soc.* 2017, **139**, 16289–16296.
2. B. M. Fung, A. K. Khitrin, K. Ermolaev, *J. Magn. Reason.* 2000, **142**, 97–101.
3. G. Kresse, J. Furthmüller, *Phys. Rev. B*, 1996, **54**, 11169–11186.
4. P. E. Blochl, *Phys. Rev. B*, 1994, **50**, 17953–17979.
5. G. Kresse, D. Joubert, *Phys. Rev. B*, 1999, **59**, 1758–1775.
6. J. P. Perdew, K. Burke, M. Ernzerhof, *Phys. Rev. Lett.*, 1996, **77**, 3865–3868.
7. J. Klimeš, D. R. Bowler, A. Michaelides, *J. Phys.: Cond. Matt.* 2010, **22**, 022201.
8. A. Togo, I. Tanaka, *Scr. Mater.* 2015, **108**, 1–5.
9. Y. Q. Cheng, L. L. Daemen, A. I. Kolesnikov, A. J. Ramirez-Cuesta, *J. Chem. Theory Comput.* 2019, **15**, 1974–1982.
10. D. Saha, S. Deng, *J. Colloid. Interf. Sci.* 2010, **348**, 615–620.
11. I. Spanopoulos, P. Xydias, C. D. Malliakas, P. N. Trikalitis, *Inorg. Chem.* 2013, **52**, 855–862.
12. T. Kajiwara, M. Higuchi, D. Watanabe, H. Higashimura, T. Yamada, H. Kitagawa, *Chem.* 2014, **20**, 15611–15617.
13. J. F. Humbeck, T. M. McDonald, X. Jing, B. M. Wiers, G. Zhu, J. R. Long, *J. Am. Chem. Soc.* 2014, **136**, 2432–2440.
14. A. J. Rieth, Y. Tulchinsky, M. Dinca, *J. Am. Chem. Soc.* 2016, **138**, 9401–9404.
15. Y. Chen, F. F. Zhang, Y. Wang, C. Y. Yang, J. F. Yang, J. P. Li, *Micropor. Mesopor Mat.* 2018,

258, 170–177.

16. A. J. Rieth, M. Dinca, *J. Am. Chem. Soc.* 2018, **140**, 3461–3466.
17. Y. Chen, X. Zhang, K. Ma, Z. Chen, X. Wang, J. Knapp, S. Alayoglu, F. Wang, Q. Xia, Z. Li, T. Islamoglu, O. K. Farha, *ACS Appl. Nano M.* 2019, **2**, 6098–6102.
18. Y. Zhang, X. Zhang, Z. Chen, K. I. Otake, G. W. Peterson, Y. Chen, X. Wang, L. R. Redfern, S. Goswami, P. Li, T. Islamoglu, B. Wang, O. K. Farha, *ChemSusChem* 2020, **13**, 1710–1714.
19. R. Cao, Z. Chen, Y. Chen, K. B. Idrees, S. L. Hanna, X. Wang, T. A. Goetjen, Q. Sun, T. Islamoglu, O. K. Farha, *ACS Appl. Mater. Inter.* 2020, **12**, 47747–47753.
20. D. W. Kim, D. W. Kang, M. Kang, J. H. Lee, J. H. Choe, Y. S. Chae, D. S. Choi, H. Yun, C. S. Hong, *Angew. Chem. Int. Ed.* 2020, **132**, 22720–22725.
21. T. Yoskamtorn, P. Zhao, X. P. Wu, K. Purchase, F. Orlandi, P. Manuel, J. Taylor, Y. Li, S. Day, L. Ye, C. C. Tang, Y. Zhao, S. C. E. Tsang, *J. Am. Chem. Soc.* 2021, **143**, 3205–3218.
22. H. G. W. Godfrey, I. Silva, L. Briggs, J. H. Carter, C. G. Morris, M. Savage, T. L. Easun, P. Manuel, C. A. Murray, C. C. Tang, M. D. Frogley, G. Cinque, S. Yang, M. Schroder, *Angew. Chem. Int. Ed.* 2018, **57**, 14778–14781.
23. X. Han, W. Lu, Y. Chen, I. Silva, J. Li, L. Lin, W. Li, A. M. Sheveleva, H. G. W. Godfrey, Z. Lu, F. Tuna, E. J. L. McInnes, Y. Cheng, L. L. Daemen, L. J. M. McPherson, S. J. Teat, M. D. Frogley, S. Rudic, P. Manuel, A. J. Cuesta, S. Yang, M. Schroder, *J. Am. Chem. Soc.* 2021, **143**, 3153–3161.
24. P. Lyu, A. Wright, A. Olvera, P. Mileo, J. Zárate, E. Ahumada, V. Martis, D. Williams, M. Dincă, I. Ibarra, and G. Maurin, *Chem, Mater.* 2021, *33*, 6186–6192.
25. C. Marsh, X. Han, J. Li, Z. Lu, S. P. Argent, I. Silva, Y. Cheng, L. L. Daemen, A. J. Cuesta, S. P. Thompson, A. J. Blake, S. Yang, M. Schroder, *J. Am. Chem. Soc.* 2021, **143**, 6586–6592.
26. E. Binaeian, Y. Li, H. Tayebi, D. Yuan, *J. Hazard. Mater.* 2021, **416**, 125933.
